# Identification and Expression Analysis of Candidate Genes Involved in Carotenoid Biosynthesis in Chickpea Seeds

**DOI:** 10.3389/fpls.2016.01867

**Published:** 2016-12-15

**Authors:** Mohammad K. Rezaei, Amit Deokar, Bunyamin Tar'an

**Affiliations:** Crop Development Centre/Department of Plant Sciences, College of Agriculture and Bioresources, University of SaskatchewanSaskatoon, SK, Canada

**Keywords:** chickpea, single nucleotide polymorphism (SNP), carotenoids, gene expression

## Abstract

Plant carotenoids have a key role in preventing various diseases in human because of their antioxidant and provitamin A properties. Chickpea is a good source of carotenoid among legumes and its diverse germplasm and genome accessibility makes it a good model for carotenogenesis studies. The structure, location, and copy numbers of genes involved in carotenoid biosynthesis were retrieved from the chickpea genome. The majority of the single nucleotide polymorphism (SNPs) within these genes across five diverse chickpea cultivars was synonymous mutation. We examined the expression of the carotenogenesis genes and their association with carotenoid concentration at different seed development stages of five chickpea cultivars. Total carotenoid concentration ranged from 22 μg g^−1^ in yellow cotyledon kabuli to 44 μg g^−1^ in green cotyledon desi at 32 days post anthesis (DPA). The majority of carotenoids in chickpea seeds consists of lutein and zeaxanthin. The expression of the selected 19 genes involved in carotenoid biosynthesis pathway showed common pattern across five cultivars with higher expression at 8 and/or 16 DPA then dropped considerably at 24 and 32 DPA. Almost all genes were up-regulated in CDC Jade cultivar. Correlation analysis between gene expression and carotenoid concentration showed that the genes involved in the primary step of carotenoid biosynthesis pathway including carotenoid desaturase and isomerase positively correlated with various carotenoid components in chickpea seeds. A negative correlation was found between hydroxylation activity and provitamin A concentration in the seeds. The highest provitamin A concentration including β-carotene and β-cryptoxanthin were found in green cotyledon chickpea cultivars.

## Introduction

Chickpea (*Cicer arietinum* L.) is one of the most important legume crops in the semi-arid tropics. Its worldwide production ranks second after common bean (FAOSTAT, 2012). It is considered as one of the most important food legumes in the developing countries because of its nutritional value and its capacity for symbiotic nitrogen fixation that can provide the entire crop demand for nitrogen (Jukanti et al., 2012).

Two chickpea types, kabuli and desi, are commonly grown. Selection for traits like flower color and zero tannins in seed resulted in the evolving kabuli from desi chickpea in the Mediterranean basin (van der Maesen, 1972; Moreno and Cubero, 1978; Jana and Singh, 1993). Chickpea has a relatively small (740 Mb) diploid (2*n* = 2*x* = 16) genome (Arumuganathan and Earle, 1991) and the genome sequence of both kabuli (Varshney et al., 2013) and desi (Jain et al., 2013) types are available that makes chickpea a good case for legume genetic and genomic research.

Chickpea is a good source of vitamins including riboflavin, niacin, thiamin, and β-carotene as the precursor of vitamin A (Cabrera et al., 2003; Abbo et al., 2005). Vitamin A deficiency leads to xerophthalmia that causes blindness among children (World Health Organization, 2009). Vitamin A deficiency also increases the chance of getting malaria and diarrheal disease (ACC/SCN, 2000). Carotenoids are categorized as a group of lipophilic yellow, orange, and red pigments primarily produced by photosynthetic organisms and also by certain fungi and bacteria (Khoo et al., 2011). They play a key role in photosynthesis and prevent photooxidation damage in plants (Howitt and Pogson, 2006). There are two major classes of carotenoids: (i) oxygenated (or xanthophyll) that includes lutein, violaxanthin, and neoxanthin and (ii) non-oxygenated (or carotenes) that include β-carotene and lycopene (DellaPenna and Pogson, 2006).

Plant carotenoids are C_40_ tetraterpenoids with conserved biosynthetic pathway that can be found in all photosynthetic tissues (DellaPenna and Pogson, 2006; Grotewold, 2006). The plastid-localized 2-C-methyl-D-erythritol 4-phosphate (MEP) pathway is responsible for producing a large number of carbon flux used for carotenoids biosynthesis (Giuliano, 2014). In the first step of MEP pathway, 1-deoxy-D-xylulose 5-phosphate (DXP) is derived from pyruvate and glyceraldehydes-3-phosphate under the control of 1-deoxy-D-xylulose-5-phophate synthase (DXS). The conversion of DXP to MEP is performed by 1-deoxy-D-xylulose 5-phosphate reductoisomerase (DXR; Julliard and Douce, 1991; Julliard, 1992). In the following steps, production of isopentenyl diphosphate (IPP) and dimethylallyl diphosphate (DMAPP) is mediated by1-hydroxy-2-methyl-2-(E)-butenyl 4-diphosphate reductase (HDR; Lichtenthaler, 1999). Then, three molecules of IPP and one molecule of DMAPP are condensed into geranyl-geranyl diphosphate (GGPP) by GGPP synthase (Kleing, 1989). Two molecules GGPPs are converted to phytoene under the control of phytoene synthase (*PSY*; Rodríguez-Concepción, 2010). Subsequently, phytoene is converted into lycopene through four desaturation and two isomerization reactions (Bartley et al., 1991; Albrecht et al., 1995; Chen et al., 2010; Yu et al., 2011).

Lycopene cyclization is facilitated by two enzymes including lycopene β-cyclase (*LCYB*) and lycopene ε-cyclase (*LCYE*) that finally produce β-carotene and α-carotene from lycopene (Pogson et al., 1996; Owens et al., 2014). The two molecules, β-carotene and α-carotene, are then converted into zeaxanthin (β, β-carotene 3,3′-diol) and lutein (β, α-carotene 3,3′-diol), respectively, by β-carotene hydroxylase (Britton, 1990). The conversion of zeaxanthin to violaxanthin is mediated by the enzyme zeaxanthin epoxidase (*ZEP*; Misra et al., 2006; Chen et al., 2014).

Carotenoids are also precursors for apocarotenoids such as plant hormone abscisic acid, which is essential for plant growth and development (Kermode, 2005; Umehara et al., 2008). Apocarotenoid formation is mediated by carotenoid cleavage dioxygenases (CCD; Auldridge et al., 2006). Violaxanthin de-epoxidase works as part of the xanthophyll (or violaxanthin) cycle and has a key role in the de-epoxidation of xanthophyll pigments such as violaxanthin (V) and antheraxanthin (A) into zeaxanthin (Z) (Misra et al., 2006; Chen et al., 2014). In the last step, violaxanthin is converted into allenic carotenoid neoxanthin by neoxanthin synthase (*NSY*; Welsch et al., 2008). The detail pathway of carotenoid biosynthesis can be seen in the report by Yan et al. (2010) and da Silva Messias et al. (2014). Each class of these enzymes seems to be responsible for the carotenoid metabolism at different and specific subcellular sites under both normal and stress conditions (Rubio et al., 2008).

Specific gene family members that are responsible for carotenoid content and composition during endosperm development have been well characterized in maize (Li et al., 2008; Vallabhaneni and Wurtzel, 2009; Vallabhaneni et al., 2009). Various approaches including metabolic and genetic engineering (Welsch et al., 2010; Kumar et al., 2012; Mintz-Oron et al., 2012; Nogueira et al., 2013; Ariizumi et al., 2014), advanced genomics and bioinformatics (Wurtzel et al., 2012), genomic-assisted selection (Campbell et al., 2014; Owens et al., 2014) and transcriptome analysis (Caroca et al., 2013; Frusciante et al., 2014) have been applied for studying carotenogenesis and improvement of carotenoid content in different crops. To develop chickpea cultivars with higher carotenoid concentration, information on the genetic basis of carotenogenesis in chickpea is substantial. The availability of genome assembly of chickpea (Jain et al., 2013; Varshney et al., 2013) provides a good source of information to identify the potential candidate genes involved in carotenogenesis.

The objectives of this research were first to identify candidate genes for carotenogenesis in chickpea through genome-wide analysis, and secondly to examine their expression pattern and their correlation with carotenoid concentration at different seed developmental stages across five diverse chickpea cultivars.

## Materials and methods

### Candidate gene analysis

The sequences of genes involved in the carotenoid pathway were collected from *Medicago truncatula* and *Arabidopsis thaliana* genomes in the GeneBank database such as National Center for Biotechnology Information (NCBI BLAST® online database). The sequences were blasted against CDC Frontier genome assembly (Varshney et al., 2013) in order to retrieve the gene sequences from chickpea. The structure of the genes and proteins in the carotenoid and isoprenoid pathways were analyzed in chickpea, *M. truncatula* and *A. thaliana*. To evaluate the similarity of the genes that have more than two copy numbers, protein sequence alignment was done using Bio Edit sequence alignment editor software (Hall, 1999).

### Re-sequencing and SNP calling

Whole genome resequencing was done on four chickpea cultivars CDC Verano, CDC 441-34, CDC Jade, and CDC Cory. The Paired-end (PE) genomic DNA libraries were constructed from 1 μg of gDNA using Illumina TruSeq DNA PCR-Free HT Library Preparation Kits (Illumina, Inc) and sequenced on Illumina HiSeq 2500 using 2 × 125 chemistry.

The raw data were subjected to filtration and correction steps using Trimmomatic v0.35 (Bolger et al., 2014). The high-quality reads obtained were then aligned to CDC Frontier reference genome sequence using BWAv0.7.12 (Li, 2013), and finally, variant calling was performed using GATK v3.5 HaplotypeCaller pipeline (McKenna et al., 2010).

We selected 32 genes from both carotenoid and isoprenoid pathways for SNP analysis. The genome assembly of CDC Frontier was considered as the reference and the sequences from the other four cultivars were compared to the CDC Frontier genome annotation. The 2 kb upstream and downstream of each gene sequence were chosen to cover the intergenic region for SNP discovery. Using SnpEff software, the SNPs among the five chickpea cultivars were identified and extracted (Cingolani et al., 2012).

### Seed sample collection

Five chickpea cultivars with different cotyledon colors including CDC Frontier (yellow cotyledon kabuli), CDC Verano (green cotyledon kabuli), CDC 441-34 (yellow cotyledon kabuli), CDC Jade (green cotyledon desi), and CDC Cory (yellow cotyledon desi) were grown in 15 L pots filled with Sunshine mix #4 media (SunGrow, Seba Beach, Alberta, Canada) in the green house. The NPK fertilizer (20-20-20) was added to each pot (three g L^−1^) for three times after plants reached the height of 20 cm.

The flowers were tagged at the day of anthesis (before the flowers open completely) and three to four developing seeds (pods) were harvested at each growth stage at 8, 16, 24, and 32 days post-anthesis (DPA). Three biological replicates were planted for each cultivar and seed growth stage. Harvested pods were immediately floated in liquid nitrogen and were kept in −80°C until RNA extraction.

### Gene expression analysis

Different primer pairs were designed using Primer3 online program (http://bioinfo.ut.ee/primer3-0.4.0/primer3/) for q-PCR analysis and their accuracy was checked by the Primer-BLAST program (Table S4).

Total RNA was isolated from chickpea seeds of four developmental stages using hexadecyltrimethylammonium bromide (CTAB) according to the procedure described by Kannan et al. (2014). Extracted RNA samples were treated with DNase I (Life Technology, Invitrogen, USA) to remove any DNA contamination. The synthesis of first cDNA strand was performed using Sensi FAST™ cDNA Synthesis Kit (BIOLINE, USA). The real-time PCR assay was conducted using C1000 Touch™ Thermal Cycler (BIO-RAD, USA) using the Sensi FAST™SYBR NO-ROX Kit (BIOLINE, USA). The relative expression was calculated using 2^(−ΔΔCt)^ method (Livak and Schmittgen, 2001). In order to find the most appropriate internal control, six housekeeping genes including *actin 1 (Act1), elongation factor 1-alpha (Ef1*α*), glyceraldehyde-3-phosphate dehydrogenase (GAPDH), initiation factor 4a (IF4a), heat shock protein 90 (HSP90)*, and *18S ribosomal RNA (18SrRNA)* (Table S4) were selected and examined for their expression (Garg et al., 2010). The 8 DPA stage of CDC Frontier was used as the reference sample and two technical replicates were used for each biological replication to minimize sampling errors. We applied UPMG method to develop a dendrogram based on K-means clustering with Cluster v3.0 program (Eisen et al., 1998). The gene expression patterns are presented as a heat map using Treeview v1.60 (Page, 1996). The gene expression levels are also presented as bar graphs (Figure S2).

### Carotenoid measurement

The seed carotenoid was measured across three developmental stages including 16, 24, and 32 DPA of five chickpea cultivars using high-performance liquid chromatography (HPLC). We used 100 mg of fine powder from whole chickpea seeds for carotenoid extraction. The samples were premixed with 400 μl of (1:1 of methanol and DCM [dichloromethane]) and then were centrifuged for 15 min at 11,000 rpm. The supernatant was transferred to a new tube and 400 μl of 100% acetonitrile was added and centrifuged for 5 min at 11,000 rpm. All solution was mixed with 0.1% BHT (butylated hydroxytoluene) to minimize carotenoid oxidation.

Chromatography was conducted using the Agilent 1200 LC system with Chemstation software (Agilent Technologies, Santa Clara, CA, USA). Separation was done on Prodigy 5 μm (250 × 4.60 mm) column with the mobile phase 58:20:22 acetonitrile/dichloromethane/methanol flowing at 0.8 ml/min. One hundred microliter volume from each sample was injected in each run for 45 min. Detection of various components was done using a photodiode array detector monitoring at a 450 nm wavelength.

Five standards including lutein and violaxanthin (ChromaDex, Irvine, CA, USA), zeaxanthin, β-carotene and β-cryptoxanthin (95% purity; Sigma-Aldrich Canada, Oakville, ON) were used to make linear standard curves as described by Ashokkumar et al. (2014). The regression coefficients for the calibration were obtained as follow: violaxanthin (*y* = 11.6*x* − 16.95, *R*^2^ = 0.992), lutein (*y* = 10.5*x* + 27.88, *R*^2^ = 0.999), zeaxanthin (*y* = 8.1*x* + 5.81, *R*^2^ = 0.995), β-cryptoxanthin (*y* = 40.02*x* + 56.2, *R*^2^ = 0.999) and β-carotene (*y* = 15*x* + 47.63, *R*^2^ = 0.999). The y denoted peak area and the *x* represented concentration (μg mL^−1^).

UV-visible spectra analysis and comparison of retention time with the authentic standard was used for carotenoid determination (Ashokkumar et al., 2014). The retention time was 3.8, 4.5, 5.6, 9.3, and 22.3 min for violaxanthin, lutein, zeaxanthin, β-cryptoxanthin and β-carotene, respectively. We used three biological replicates with two injections as technical repeats to improve the analysis by the HPLC. Results were converted into μg g^−1^ as carotenoid concentration.

### Statistical analysis

Pearson correlation analysis was done between transcript levels and carotenoid concentrations in chickpea seeds at different developmental stages. All the statistical analyses were done using SAS software (Version 9.1, SAS Institute Inc., Cary, NC, USA).

## Results

### Carotenoid biosynthesis genes in chickpea, Arabidopsis, and Medicago

The total number of genes for both isoprenoid and carotenoid pathways were 32 in chickpea and 26 in Arabidopsis. We found more copy numbers of some genes from both pathways in chickpea compared to Arabidopsis which can be explained by the differences in the genome size of these two species. Chickpea genome size of 740 Mbp (Varshney et al., 2013) is over five times larger than Arabidopsis (135 Mbp; Arabidopsis Genome Initiative, 2000).

The properties of carotenoid and isoprenoid genes in *M. truncatula* were similar to chickpea as both species share common legume family. Except for three genes, *PSY, BCH*, and *ZEP*, we found similar copy numbers for the rest of the carotenoid and isoprenoid genes in *M. truncatula* and in chickpea (Table S1). The estimated genome size of *M. truncatula* is 465 Mbp that is bigger than that of *Arabidopsis* (Bennett and Leitch, 2011).

In chickpea, the genes *CCD1* and *GGPPS2* represent the largest and smallest size with 15 Kbp and 1.8 Kbp in size, respectively. Four genes including *GGPPS1, GGPPS2, LCYB*, and *NSY* had only one exon, whereas ZEP1 and ZEP2 had the highest number of exons (16 exons). In Arabidopsis, the largest and smallest genes were PDS with 6.6 Kbp and *GGPPS2* with 1.7 Kbp, respectively. Two genes GGPPS1 and GGPPS2 had only one exon, while *ZEP1* was detected to have the highest number of exons (16 exons) in Arabidopsis (Table S1).

Domain analysis showed that the largest number of genes in the pathways have the Rossmann-fold NAD(P)H/NAD(P)(+) binding (NADB) domain in their structure. The main domain in *PSY* genes is Isoprenoid Biosynthesis Enzymes-Class 1 that is similar to *GGPPS* genes from isoprenoid pathway as observed in Arabidopsis and Medicago (data not shown). The properties of all the domains are listed in Supplemental information (Table S2).

Two genes including *DXS* and *PSY* each with four copy numbers were chosen for sequence similarity analysis in chickpea. Sequence alignment and similarity matrix indicated that *DXS1* and *DXS2* had the highest similarity (0.842) and *DXS4* had the lowest level of similarity with other DXS genes. In case of *PSY* genes, *PSY2*, and *PSY3* were highly similar (0.816) and *PSY1* and *PSY4* had the lowest similarity (0.484) within this group (Figure S1, Table S3).

### Sequence analysis and SNP identification

**Figure 1:**
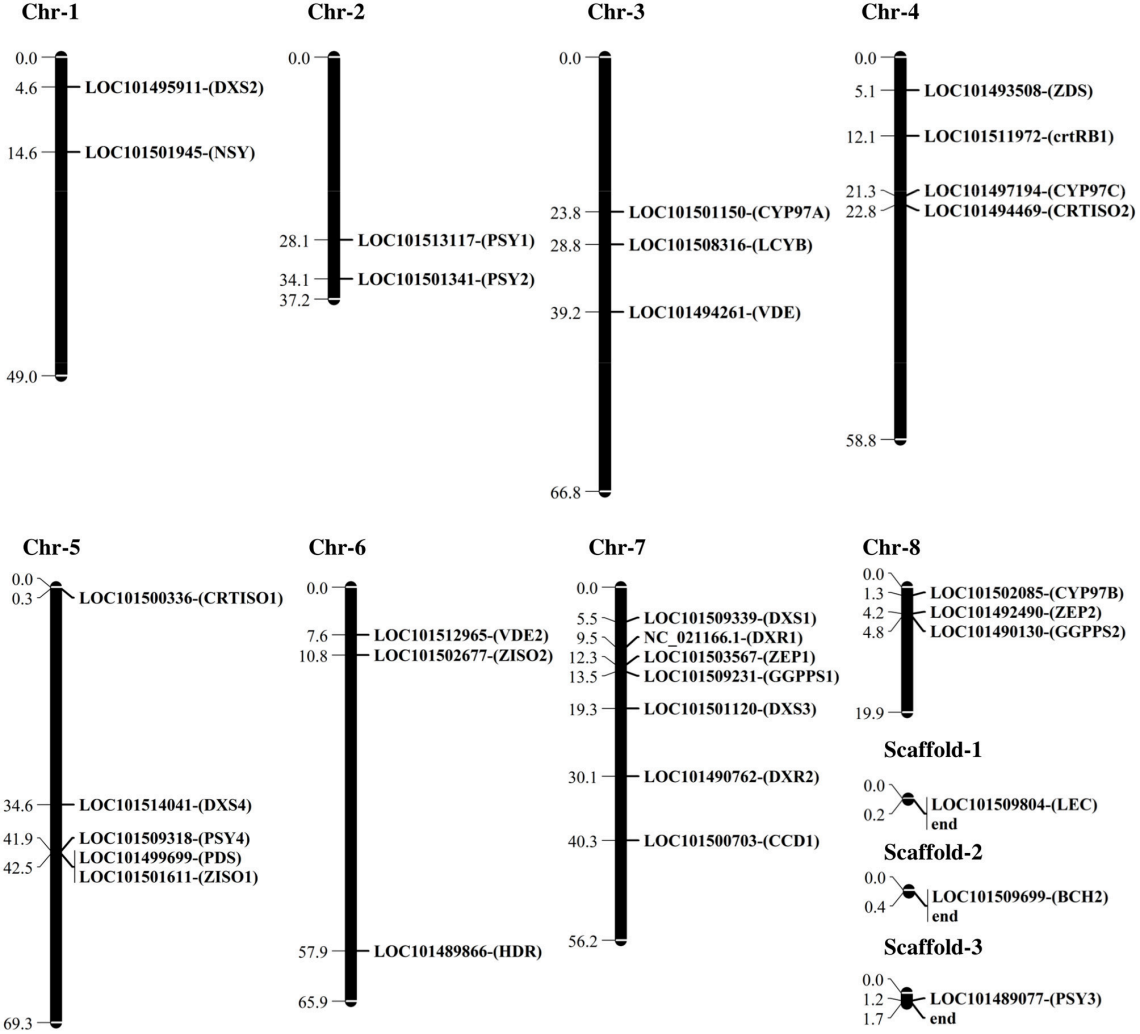
**Physical map (Mbp) of chickpea carotenogenesis genes including ***DXS1 and DXS2***, ***1-deoxy-D-xylulose-5-phosphate synthase; DXR1 and DXR2, 1-deoxy-D-xylulose 5-phosphate reductoisomerase; HDR, 1-hydroxy-2-methyl-2-(E)-butenyl 4-diphosphate reductase; GGPPS1 and GGPPS2, geranyl-geranyl diphosphate synthase; PSY1, PSY2, PSY3, and PSY4, phytoene synthase; PDS, Phytoene desaturase synthase; ZISO1 and ZISO2, 15-cis-zeta-carotene isomerase; ZDS***, ζ-***carotene desaturase; CRTISO1 and CRTISO2, prolycopene isomerase; LBC, lycopene*** β-***cyclase; LCY***ε, ***lycopene*** ε-***cyclase; BCH1 and BCH2***, β-***carotene hydroxylase; CYP97A, cytochrome P450-type*** β-***hydroxylase; CYP97B, cytochrome P450-type 97B; CYP97C, cytochrome P450-type monooxygenase; ZEP1 and ZEP2, zeaxanthin epoxidase; VDE, violaxanthin de-epoxidase; CCD1, crotenoid 9,10(9***′,***10***′***)-cleavage dioxygenase 1; and NSY, neoxanthin synthase*** with their name, accession numbers, and chromosomal location (Ca) that were analyzed in this study**. The genes in this figure were retrieved from the chickpea genome assemblies of both kabuli (Varshney et al., 2013) and desi (Jain et al., 2013) types.

Sequence analysis was done on the 32 candidate genes (Figure 1) that play an important role in carotenoid biosynthesis. In total 476 SNPs were found in the upstream, exon, intron and downstream sequences of the genes across the five chickpea cultivars. A total of 17 SNPs (Table 1) was found in the coding region of *1-deoxy-D-xylulose-5-phosphate synthase* (*DXS1* and *DXS2*), *lycopene* β-*cyclase* (*LBC*), β-*carotene hydroxylase* (*BCH1*), *zeta carotene isomerase* (*ZISO2*), *1-deoxy-D-xylulose 5-phosphate reductoisomerase* (*DXR2*), and *geranyl-geranyl diphosphate synthase* (*GGPPS2*). Only two missense mutations, which code for different amino acid, were found in *ZISO2*, while the rest of the SNPs were synonymous mutation. Consequently, the amino acid Serine (S) changed to Proline (P) at position 15 and Phenylalanine (F) to Leucine (L) at position 147 in CDC Verano compared to the reference genome CDC Frontier.

**Table 1:** **The list of 17 SNPs found in the coding region of ***DXS1 and DXS2, 1-deoxy-D-xylulose-5-phosphate synthase; LBC, lycopene*** β-***cyclase; BCH1***, β-***carotene hydroxylase; ZISO2, zeta carotene isomerase; DXR2, 1-deoxy-D-xylulose 5-phosphate reductoisomerase; and GGPPS2, geranyl-geranyl diphosphate synthase*** with their chromosomal location, physical position (bp) and the differences between the reference (CDC Frontier) and the alternative across four other cultivars**.

**Gene**	**Chromosome**	**Position (bp)**	**Reference**	**Alternative**	**CDC 441-34**	**CDC Jade**	**CDC Cory**	**CDC Verano**
*DXS2*	Ca1	4631533	G	A	1/1	1/1	1/1	1/1
*LBC*	Ca3	28752373	A	G	1/1	1/1	1/1	1/1
*BCH1*	Ca4	12093051	T	C	0/0	1/1	0/1	0/1
*ZISO2*	Ca6	10805615	C	T	0/0	0/0	0/0	0/1
*ZISO2*	Ca6	10805725	T	G	0/0	0/0	0/0	0/1
*ZISO2*	Ca6	10805905	C	A	0/0	0/0	0/0	0/1
*ZISO2*	Ca6	10805923	C	T	0/0	0/0	0/0	0/1
*DXS1*	Ca7	5529110	A	G	0/0	1/1	1/1	0/1
*DXS1*	Ca7	5529191	A	G	0/0	1/1	1/1	0/0
*DXR2*	Ca7	30117254	T	C	1/1	1/1	0/0	1/1
*DXR2*	Ca7	30117353	A	G	1/1	1/1	0/1	1/1
*DXR2*	Ca7	30117362	A	G	1/1	1/1	0/1	1/1
*DXR2*	Ca7	30117491	C	T	1/1	1/1	0/1	1/1
*DXR2*	Ca7	30117821	T	C	1/1	1/1	0/1	1/1
*DXR2*	Ca7	30136051	A	G	1/1	1/1	0/0	1/1
DXR2	Ca7	30136328	G	A	1/1	1/1	0/1	1/1
GGPPS2	Ca8	4790033	C	T	0/0	0/1	0/0	0/1

### Carotenoid concentration

The highest concentration of lutein was observed in CDC Jade at 16 DPA. The lutein concentration dropped significantly at 24 and 32 DPA. The lowest concentration of lutein was observed in CDC Frontier. The concentration of zeaxanthin was highest in CDC Cory followed by CDC Jade. The zeaxanthin concentration was lowest and similar in three cultivars CDC Frontier, CDC 441-34 and CDC Verano (Table 2). The highest concentration of β-carotene was observed in CDC Cory and CDC Jade at 16 DPA, but at the later stage, we observed the highest concentration in CDC Jade and CDC Verano. CDC Jade and CDC 441-34 showed the highest and lowest concentration of β-cryptoxanthin, respectively, in almost all stages. In addition, the highest and lowest concentration of violaxanthin was observed in CDC Jade and CDC Frontier, respectively. Total carotenoid was highest in CDC Jade and lowest in CDC Frontier. In general, the concentration of different types of carotenoid decreased from 16 to 32 DPA in all cultivars. Overall in the five cultivars, the concentration of lutein was the highest followed by zeaxanthin, β-carotene, β-cryptoxanthin and violaxanthin (Table 2).

**Table 2:** **Concentration (μg g^**−1**^) of different seed carotenoids including Lutein, Zeaxanthin, β-carotene, β-cryptoxanthin, Violaxanthin and total carotenoid ± Se in five chickpea cultivars (CDC Frontier, CDC 441-34, CDC Verano, CDC Cory, and CDC Jade) at 16, 24, and 32 days post anthesis (DPA)**.

**Cultivars**	**DPA**	**Lutein**	**Zeaxanthin**	**β-Carotene**	**β-Cryptoxanthin**	**Violaxanthin**	**Total**
CDC Frontier	16	26.5±1.01	18.25±0.19	2.15±0.16	2.15±0.01	1.32±0.01	50.37
CDC Frontier	24	15.4±0.08	10.1±0.07	1.73±0.19	0.94±0.03	0.66±0.03	28.85
CDC Frontier	32	11.71±0.14	8.74±0.19	1.68±0.14	0.79±0.15	0.68±0.15	23.64
CDC 441-34	16	27.43±1.82	20.33±0.68	1.92±0.3	1.18±0.18	1.8±0.18	52.69
CDC 441-34	24	17.86±0.35	14.44±0.19	1.86±0.15	0.98±0.0	1.48±0.01	36.63
CDC 441-34	32	10.64±0.81	8.1±0.33	1.74±0.08	0.94±0.01	0.68±0.01	22.12
CDC Verano	16	37.43±1.09	29.51±0.16	2.25±0.08	2.3±0.15	2.1±0.15	73.59
CDC Verano	24	19.01±0.45	19.82±0.06	1.78±0.14	1.0±0.02	1.1±0.02	42.37
CDC Verano	32	17.98±0.44	9.05±0.09	1.9±0.02	0.82±0.13	1.05±0.13	30.83
CDC Cory	16	37.36±0.36	31.84±0.6	2.37±0.07	2.55±0.12	2.04±0.12	76.18
CDC Cory	24	22.36±0.14	22.16±0.19	1.89±0.0	1.43±0.0	0.99±0.01	48.84
CDC Cory	32	17.72±0.18	18.51±0.05	1.76±0.14	0.86±0.07	0.67±0.07	39.54
CDC Jade	16	45.93±0.64	26.12±0.86	2.61±0.06	2.88±0.13	2.51±0.13	80.07
CDC Jade	24	31.82±0.14	17.89±0.38	2.43±0.02	2.54±0.39	1.76±0.39	56.44
CDC Jade	32	24.09±0.34	14.76±0.11	2.13±0.01	1.74±0.04	1.28±0.04	44.03

### Gene expression

One of the most important factors for the real-time PCR data analysis is finding a good internal control because all the results would be normalized based on the cycle threshold (CT) of the house-keeping gene. Among all the house-keeping genes tested in this study, the 18SrRNA showed constant expression within and among the cultivars at different developmental stages (data not shown); therefore, it was used as the internal control for the expression analysis.

**Figure 2:**
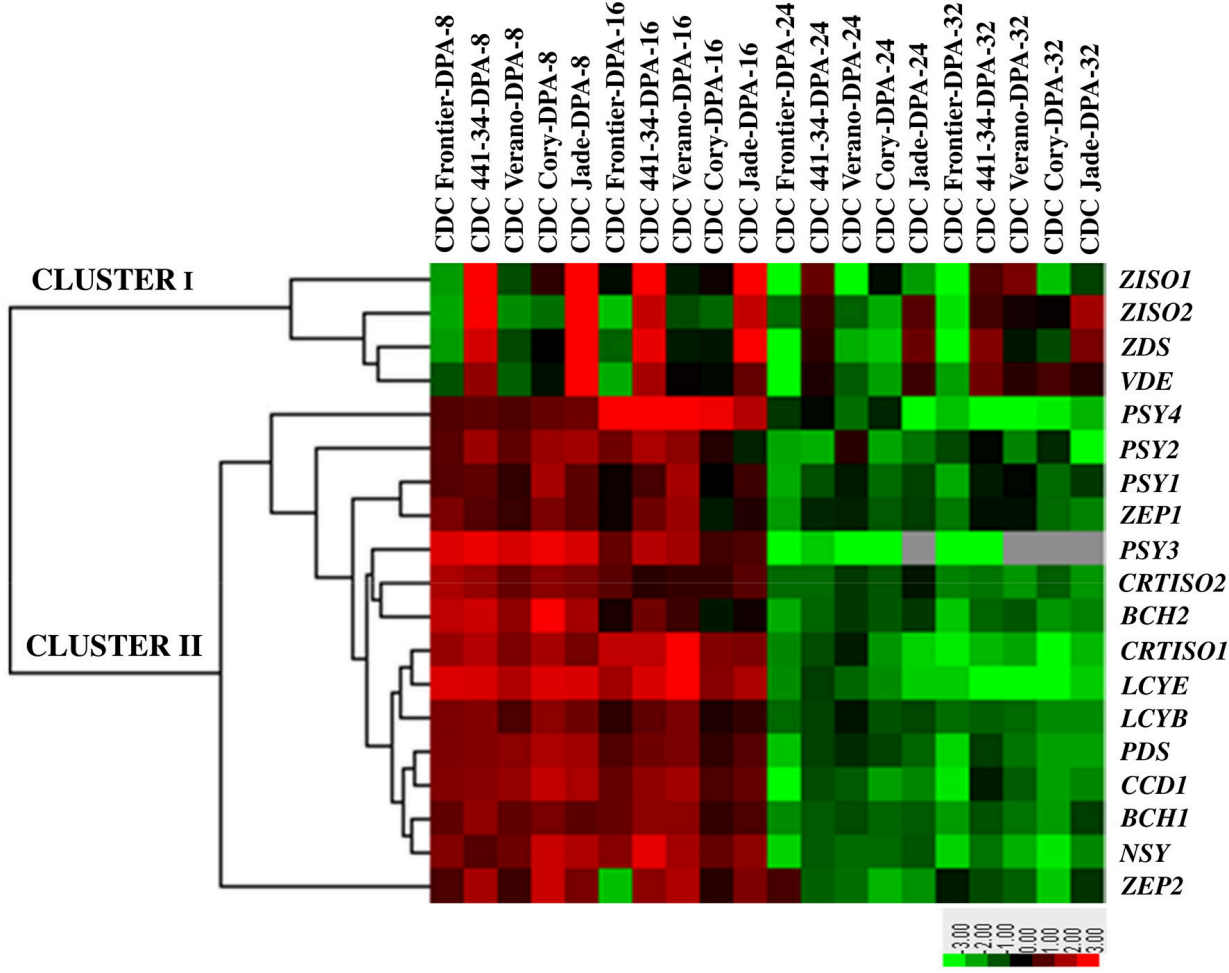
**Heat map of the expression pattern of the carotenogenesis genes in chickpea seeds at four developmental stages 8, 16, 24, and 32 days post anthesis (DPA) of five cultivars CDC Frontier, CDC 441-34, CDC Verano, CDC Cory, and CDC Jade**. The list of the genes includes *PSY1, Phytoene synthase 1; PSY2, Phytoene synthase 2; PSY3, Phytoene synthase 3; PSY4, Phytoene synthase 4; PDS, Phytoene desaturase synthase, ZISO1, 15-cis-zeta-carotene isomerase 1; ZISO2, 15-cis-zeta-carotene isomerase 1; ZDS*, ζ-*carotene desaturase, CRTISO1, carotene isomerase; CRTISO2, carotene isomerase 2; LCYB, lycopene* β-*cyclase; LCYE, lycopene* ε-*cyclase; BCH1*, β-*carotene hydroxylase 1; BCH2*, β-*carotene hydroxylase 2; ZEP1, zeaxanthin epoxidase 1; ZEP2, zeaxanthin epoxidase2; CCD1, crotenoid 9,10(9',10')-cleavage dioxygenase 1; VDE, violaxanthin de-epoxidase;* and *NSY, neoxanthin synthase*. Up-regulation and down-regulation are indicated in red and green colors, respectively. No detected expression is indicated with gray color.

The expression of four genes *ZISO1, ZISO2, ZDS*, and *VDE* were grouped in cluster I and the rest of carotenoid genes were included in cluster II (Figure 2). The expression level of phytoene synthase 1 was higher at early stage (8 DPA) than in the later stages of all cultivars. The highest and the lowest transcript levels of phytoene synthase 1 were found in CDC Jade and CDC Frontier, respectively. The same pattern was also observed for phytoene synthase 2 and 3. In contrast, phytoene synthase 4 had the lowest expression level in CDC Jade. The expression of phytoene desaturase was dominant at 8 DPA and its expression level was almost similar in all cultivars. Significant expression of carotene 15-*cis*-ζ-carotene isomerase 1 and 2 and ζ-carotene desaturase was obtained in CDC Jade and CDC 441-34 cultivars at 8 DPA (Figure 2 and Figure S2).

The next two enzymes in the pathway, carotene isomerase (*CRTISO*) 1 and 2, were expressed predominantly in the kabuli type. Lycopene β-cyclase, lycopene ε-cyclase, and carotenoid dioxygenase were highly expressed at 8 DPA in all cultivars. On average the lowest expression of β-carotene hydroxylase 1 and 2 was observed in CDC Jade and CDC Verano cultivars at 8 DPA. Expression of violaxanthin de-epoxidase was higher in CDC Jade at 8 DPA than that of other cultivars and the lowest expression was observed in CDC Frontier. Expression pattern of the two zeaxanthin epoxidase was similar with higher expression at 8 DPA, except for CDC Verano in which its expression increased at 16 DPA. The highest expression of neoxanthin synthase was observed in CDC 441-34 at 16 DPA (Figure 2 and Figure S2).

### Correlation analysis

Correlation analysis revealed positive correlations between the expression of the genes in the primary steps of the carotenogenesis including zeta-carotene desaturase, zeta carotene isomerase, poly lycopene isomerase, and carotenoid concentration. A negative correlation was obtained between hydroxylation and cleavage activities and provitamin A concentration (Table 3).

**Table 3:** **Pearson correlation analysis between transcript levels of the genes involved in carotenoid biosynthesis pathway and their products in chickpea seed at different days post anthesis (DPA)**.

**Gene**	**Carotenoid type**	**Stage**	**Correlation coefficient**
Zeta carotene isomerase 2	Lutein	32 DPA	0.90^*^
Zeta carotene isomerase 2	Zeaxanthin	32 DPA	0.86^*^
Zeta carotene isomerase 2	β-Carotene	32 DPA	0.94^**^
Zeta carotene isomerase 2	β-Cryptoxanthin	32 DPA	0.93^**^
Zeta carotene isomerase 2	Total Carotenoid	32 DPA	0.90^*^
Neoxanthin synthase	Total	8 DPA	0.87^*^

P < 0.05 and P < 0.01 were considered for significant (^*^) and highly significant (^**^), respectively.

## Discussion

The current study revealed that the majority of the SNPs within the genes involved in carotenoid biosynthesis resulted from synonymous substitutions. Similar patterns of variation were also found in genes of carotenoid biosynthesis in tomato, citrus, pepper, and carrot (Livingstone and Anderson, 2009). The highly conserved carotenoid biosynthesis genes across different species reflect that these genes are required for a wide range of end products essential for the plants. Any changes on these enzymes could have major deleterious effect on plant fitness. Traditionally, researchers mainly focused on the non-synonymous mutations that can result in the changing of the amino acids and consequently protein function (Sauna and Kimchi-Sarfaty, 2011). However, recent studies indicated that the synonymous mutations may have an effect on mRNA splicing (Pagani et al., 2005), mRNA stability (Kimchi-Sarfaty et al., 2007), the efficiency of protein translation, and protein folding (Sauna and Kimchi-Sarfaty, 2011). Protein expression can also be influenced by synonymous SNPs as they are involved in regulating microRNA-mediated genes (Wang et al., 2015). Therefore, the synonymous SNPs identified in the diverse chickpea cultivars in this study may have potential functional significance in carotenoid biosynthesis. Further study might be needed to examine the detail of this mechanism.

The two non-synonymous SNPs in *ZISO2* in CDC Verano are good examples of mutations that changed the amino acids as a result. The enzyme *ZISO2* is involved in isomerization activities in the carotenoid pathway. Its expression significantly correlated with various carotenoid concentrations. The green cotyledon kabuli, CDC Verano, has the highest carotenoid concentration among all the kabuli type which could be due to the mutations in *ZISO2* in this cultivar.

Variation of exon numbers among different copies of the carotenoid genes is interesting in this study. For example, among the different copies of the *CRTISO* gene the exon numbers varied from 5 to 13, while in *PSY* the exons varied from five to nine (Table S1). Exon-intron architecture is one of the mysterious issues in gene evolution (Zhu et al., 2009). It seems that the length and CG content of first exon and intron have an association with functional element in higher organism (Kalari et al., 2006). Kreimer and Pe'er (2013) also discussed that exon variant can affect gene expression. It is possible that the changes in gene expression between different copy numbers of a carotenoid gene like *PSY* in chickpea might be affected by the variation in exon number and size.

The 32 candidate genes involved in isoprenoid and carotenoid pathways were distributed across all 8 chromosomes of chickpea. Different copy numbers for some candidate genes exist in the chickpea genome (Figure 1). The *PSY* gene has been considered as a key regulator of the carotenoid pathway (Li et al., 2007, 2008). In domesticated maize, the *PSY1* locus has been the target of selective sweep and it was reported that 6.6–27.2% variations of the seed carotenoid concentration are associated with the activity of this enzyme (Palaisa et al., 2004; Chander et al., 2008; Bai et al., 2009). Pozniak et al. (2007) showed that *PSY1* is co-segregated with the 7B QTL in durum wheat (*Triticum turgidum* L. var *durum*) and confirmed the correlation of the gene with phenotypic variation for endosperm color. Cereals commonly have three homologs of *PSY* in their genome. Study on *PSY* family in maize showed that the expression of each member can be different among various tissues. Usually, the expression of *PSY1* is higher in leaves and yellow endosperm, but *PSY2* expression is significant in almost all tissues (Gallagher et al., 2004). The third family member, *PSY3* is normally expressed in embryo and root especially under stress condition (Li et al., 2007). In chickpea, we found four members of *PSY* family, which may have a positive effect on carotenoid concentration across different cotyledon colors.

Significant and positive correlation between isomerization activities and carotenoid concentration (Table 3) in chickpea indicated the key role of these enzymes in providing common precursor lycopene for carotenoid types. We also found that desaturation reaction had a positive correlation (tend to be significant) with carotenoid concentration (data not shown). In addition, the transcript levels of isoprenoid genes including 1-deoxy-D-xylulose-5-phosphate synthase (DXS3), 1-deoxy-D-xylulose 5-phosphate reductoisomerase (DXR), 1-hydroxy-2-methyl-2-(E)-butenyl 4-diphosphate reductase (HDR), geranylgeranyl diphosphate synthase (GGPS1) (Julliard and Douce, 1991; Julliard, 1992; Lichtenthaler, 1999), can significantly affect carotenoid concentration in plant (Vallabhaneni and Wurtzel, 2009), as carotenoid biosynthesis is a derivative of isoprenoid pathway (Cuttriss et al., 2011). The deficiency of ζ-carotene desaturase (*ZDS*) gene in sunflower (*Helianthus Annuus* L.) resulted in the accumulation of ζ-carotene and the absence of β-carotene, lutein and violaxanthin in cotyledon (Conti et al., 2004). In many cases, genetic transformation or overexpression of phytoene synthase and desaturase to crop plants has resulted in higher provitamin A and total carotenoid concentration (Ye et al., 2000; Paine et al., 2005; Diretto et al., 2007; Naqvi et al., 2009; Kim et al., 2012). This study showed that CDC Jade cultivar with highest carotenoid concentration also had the highest expression level of *ZDS*.

The β-carotene hydroxylation converts provitamin A compounds into xanthophylls that have no provitamin A properties (Quinlan et al., 2012), so the challenge for breeders to develop cultivars with higher provitamin A concentration is limiting the β-carotene hydroxylation (da Silva Messias et al., 2014). Diretto et al. (2006) showed that silencing of β-carotene hydroxylase results in a higher β-carotene concentration in potato. In this study, we observed lower hydroxylation activities in CDC Verano and CDC Jade cultivars with high carotenoid concentration.

Cleavage activity is involved in apocarotenoid production that consequently reduces the carotenoid concentration (Auldridge et al., 2006). Also, the carotenoid cleavage dioxygenase has been considered as a negative regulator for β-carotene concentration in *Arabidopsis* and its expression had a direct effect on the degradation and turnover of carotenoids in seeds during maturity period (Rodríguez-Ávila et al., 2011; Gonzalez-Jorge et al., 2013). For example, functional analysis of carotenoid cleavage dioxygenase mutant in *Arabidopsis* (*AtCCD1*), showed higher carotenoid concentration in mature seeds of mutant cultivar than the wild type (Auldridge et al., 2006). The important effect of *ZEP1* region on carotenoid concentration in 281 maize lines with various kernel colors has been discussed by Owens et al. (2014). It seems that the ratio between carotenoid production and its conversion into apocarotenoid, plays a key role in carotenoid concentration in chickpea. In CDC Frontier and CDC 441-34 cultivars this ratio is lower than CDC Cory and CDC Jade cultivars with higher carotenoid concentration.

Based on our results the carotenoid levels decreased from earlier stage to later stage in chickpea seeds. The ABA concentration increases during seed development and it is positively correlated with embryo maturation (Wang et al., 1998). The legume seeds, which are mostly embryo, have a significant concentration of ABA (Goldberg et al., 1994). ABA is not the only cleavage product of carotenoids and there are more products like strigolactone (Booker et al., 2004). This process can explain the reduction of carotenoid during seed development. In addition, non-enzymatic activities including oxidative stress and lipid peroxidation involved in carotenoid degradation during seed desiccation (Sattler et al., 2004, 2006; Mène-Saffrané et al., 2010). However, our knowledge regarding regulatory mechanism involved in carotenoid concentration and composition may not be complete as potential other mechanisms may occur (Shumskaya and Wurtzel, 2013; De Moura et al., 2013).

The two stay-green cultivars, CDC Jade and CDC Verano had the highest concentration of β-carotene among the Desi and the Kabuli types, respectively. Also, total carotenoid concentration is higher in pea and chickpea cultivars with green cotyledon color compared to cultivars with yellow cotyledons (Ashokkumar et al., 2014), and the same results were obtained as the case of lutein in pea (Holasovál et al., 2009).

Mutation on the *stay-green* gene (SGR) that involves in degradation of chlorophyll resulted in delayed senescence and consequently green cotyledon chickpea seed. In thylakoid membranes, carotenoids are in a complex of chlorophyll and protein of Photosystem I and II. For example, the PSI contains high β-carotene concentration and PSII is rich in lutein. In addition, the high correlation was found between lutein and chlorophyll concentration in green pea (Holasovál et al., 2009). Importantly the increased levels of carotenoid products normally associated with both the chloroplastic and the cytosolic pathways, including mevalonate, sterols, and squalene, as well as triacyl- glycerides (Kumar et al., 2012). For instance, *PSY* has a coexpression with genes involve in the synthesis of plastoquinone, NAD(P)H dehydrogenase, tiorredoxin, plastocianin, and ferredoxin (Meier et al., 2011). It seems that carotenoids in the complex of green tissues stay more stable than tissues with chlorophyll degradation. It is discussed by Ashokkumar et al. (2014) that higher lycopene cyclase activity is the main reason for higher total carotenoid in green cotyledon pea; however the cyclase activity in two green cotyledon chickpea was not higher than the other cultivars in this study. We believed that lower cleavage and hydroxylation activities have a significant effect on carotenoid concentration in green chickpea.

The total carotenoid concentration in chickpea dry seeds (9.08 μg g^−1^; Abbo et al., 2005) is higher than that genetically-engineered “golden rice” endosperm (1.6 μg g^−1^; Ye et al., 2000), or red colored wheat (1.8–5.8 μg g^−1^; Kruger and Reed, 1988; United States Department of Agriculture, 2010). However, in Golden Rice2 the carotenoid concentration increased up to 23-fold (37 μg g^−1^ dry weight) compared to the original one (Paine et al., 2005). Carotenoid concentration is a trait with high heritability that mostly influenced by genetic (cultivar), and the effect of environment is not substantial for all carotenoid types (Abbo et al., 2010; Owens et al., 2014). Lutein is the main carotenoid type in chickpea (Abbo et al., 2005, 2010) and wheat (Ramachandran et al., 2010). Desi chickpea usually has higher lutein concentration than the Kabuli type likely due to higher grain weight in Kabuli type (Ashokkumar et al., 2015). It seems that the chromosomal linkage and pleiotropy can address the association between higher lutein concentration and low seed weight (Abbo et al., 2005). The carotenoid concentration obtained in our work was consistent with results from earlier studies for Kabuli and Desi chickpeas (Ashokkumar et al., 2014, 2015).

It was reported that carotenogenesis genes are active in photosynthetic organs under various light qualities and the levels of both chlorophyll and carotenoids will increase dramatically in de-etiolation period (Romer and Fraser, 2005; Toledo-Ortiz et al., 2010). In addition, the total concentration of xanthophylls in different plant species increases dramatically in a strong light condition that results in decreasing the ratio between “lutein (L) and the xanthophylls-cycle components, zeaxanthin, antheraxanthin, and violaxanthin (Z+A+V)” (Hirschberg, 2001). In order to control the light effect on carotenoid types, we grew all plants in the green house under controlled condition.

In conclusion, the carotenoid concentration in chickpea is under the control of various genes that are associated with the structure and function of the genes in the carotenoid pathway. New technology in genome sequencing has helped us to understand the details regarding the structure, polymorphism, copy number, and location of the genes involved in carotenoid biosynthesis in chickpea seeds. Along with genome sequence data, the variability of the expression pattern and carotenoid concentration in five cultivars revealed a logical relationship between genotypes and phenotypes in this study. We demonstrated that synonymous mutations may have functional effects on the expression pattern of the different genes involved in the carotenoid biosynthesis pathway.

## Author contributions

MR conducted the experiments, analyzed, and summarized the results. MR, AD, and BT wrote and finalized the manuscript; BT conceived and directed the project.

### Conflict of interest statement

The authors declare that the research was conducted in the absence of any commercial or financial relationships that could be construed as a potential conflict of interest.

## Abbreviations

DPA: Days post anthesis.
