# Supplementary material for: Identification and Expression Analysis of Candidate Genes Involved in Carotenoid Biosynthesis in Chickpea Seeds
Source: Front Plant Sci. 2016 Dec 15;7:1867. doi: 10.3389/fpls.2016.01867 (PMC5157839; doi:10.3389/fpls.2016.01867)
Supplement: Supplementary file 1 [file DataSheet1.docx]

Table S1: List and name of the genes involved in isoprenoid and carotenoid pathways with their accessions, copy numbers, exons numbers and size in chickpea, *Arabidopsis* and *Medicago truncatula*

|  | **Chickpea** | | | **Arabidopsis** | | | **Medicago** | | |
| --- | --- | --- | --- | --- | --- | --- | --- | --- | --- |
| Gene | Accessions | Exon no | Size (Kbp) | Accession | Exon no | Size (Kbp) | Accession | Exon no | Size (Kbp) |
| DXS1 | LOC101509339 | 10 | 5.5 | AT3G21500 | 9 | 3.9 | MTR_4g118640 | 9 | 5.7 |
| DXS2 | LOC101495911 | 11 | 8.3 | AT4G15560 | 9 | 4.8 | MTR_2g020590 | 10 | 9.4 |
| DXS3 | LOC101501120 | 10 | 6 | AT5G11380 | 10 | 4.5 | MTR_8g068265 | 9 | 6.4 |
| DXS4 | LOC101514041 | 10 | 9.2 | - | - | - | MTR_3g107740 | 8 | 7.9 |
| DXR1 | LOC101494630 | 12 | 6.1 | AT5G62790 | 13 | 4.3 | MTR_4g106870 | 12 | 6.5 |
| DXR2 | LOC101490762 | 13 | 12 | - | - | - | MTR_8g012565 | 12 | 7.5 |
| HDR1 | LOC101489866 | 10 | 4.4 | AT4G34350 | 10 | 3.4 | MTR_4g069030 | 10 | 4.7 |
| HDR2 | - | - | - | - | - | - | MTR_4g069070 | 10 | 5.4 |
| GGPPS1 | LOC101509231 | 1 | 2.4 | AT4G36810 | 1 | 1.9 | MTR_8g078070 | 1 | 2.4 |
| GGPPS2 | LOC101490130 | 1 | 1.8 | AT2G23800 | 1 | 1.7 | MTR_5g019460 | 1 | 1.6 |
| PSY1 | LOC101513117 | 9 | 5.5 | AT5G17230 | 9 | 4.3 | MTR_5g076620 | 7 | 5.6 |
| PSY2 | LOC101501341 | 5 | 3.5 | - | - | - | MTR_5g012060 | 2 | 4.7 |
| PSY3 | LOC101489077 | 5 | 2.5 | - | - | - | MTR_5g090780 | 5 | 13 |
| PSY4 | LOC101509318 | 6 | 3 | - | - | - | - | - | - |
| PDS | LOC101499699 | 13 | 10 | AT4G14210 | 15 | 6.6 | MTR_5g042243 | 3 | 0.94 |
| ZISO1 | LOC101501611 | 4 | 2.8 | AT1G10830 | 4 | 2.5 | MTR_8g097190 | 4 | 4.3 |
| ZISO2 | LOC101502677 | 4 | 3.2 | - | - | - | MTR_3g084950 | 4 | 3.6 |
| ZDS | LOC101493508 | 15 | 7.6 | AT3G04870 | 14 | 4.9 | MTR_1g081290 | 14 | 7.4 |
| CRTISO1 | LOC101500336 | 5 | 3 | AT1G06820 | 13 | 4.3 | MTR_4g134780 | 5 | 3.1 |
| CRTISO2 | LOC101494469 | 13 | 8.2 | AT1G57770 | 10 | 4 | MTR_1g054965 | 14 | 7.7 |
| LCYB | LOC101508316 | 1 | 2.5 | AT3G10230 | 2 | 2.6 | MTR_7g090150 | 1 | 1.9 |
| LCYE | LOC101509804 | 11 | 8.9 | AT5G57030 | 11 | 3.6 | MTR_2g040060 | 11 | 9.3 |
| BCH1 | LOC101511972 | 7 | 3.1 | AT4G25700 | 7 | 2.6 | - | - | - |
| BCH2 | LOC101509699 | 7 | 6.1 | AT5G52570 | 7 | 2.9 | MTR_6g048440 | 7 | 6.8 |
| CYP97A | LOC101501150 | 15 | 8.3 | AT1G31800 | 15 | 4.3 | MTR_7g079440 | 15 | 9.3 |
| CYP97B | LOC101502085 | 15 | 10 | AT4G15110 | 14 | 4.3 | MTR_5g009110 | 14 | 11 |
| CYP97C | LOC101497194 | 9 | 7.4 | AT3G53130 | 12 | 6.2 | MTR_1g062190 | 9 | 7.9 |
| CCD1 | LOC101500703 | 14 | 15 | AT3G63520 | 14 | 4.1 | MTR_8g037315 | 14 | 8.4 |
| ZEP1 | LOC101503567 | 16 | 8.6 | AT5G67030 | 16 | 4.8 | MTR_5g017350 | 15 | 12 |
| ZEP2 | LOC101492490 | 16 | 7.9 | - | - | - | - | - | - |
| VDE1 | LOC101494261 | 5 | 3.9 | AT1G08550 | 7 | 3.4 | MTR_7g116630 | 6 | 4.9 |
| VDE2 | LOC101512965 | 3 | 7.5 | AT2G21860 | 2 | 2.3 | MTR_8g092050 | 2 | 4.6 |
| NSY | LOC101501945 | 1 | 2.2 | AT1G67080 | 6 | 2.1 | MTR_7g007280 | 6 | 4.2 |

Table S2: List of main domains of important proteins in isoprenoid and carotenoid pathways with their accessions in chickpea

| **Gene** | **Accession** | **Super family N treminal** | **Super family C treminal** |
| --- | --- | --- | --- |
| DXS1 | LOC101509339 | Thiamine pyrophosphate | Transketolase |
| DXS2 | LOC101495911 | Thiamine pyrophosphate | Transketolase |
| DXS3 | LOC101501120 | Thiamine pyrophosphate | Transketolase |
| DXS4 | LOC101514041 | Thiamine pyrophosphate | Transketolase |
| HDR | LOC101489866 | 4-hydroxy-3-methylbut-2-enyl diphosphate reductase (lytB_ispH) | - |
| GGPPS1 | LOC101509231 | Isoprenoid Biosynthesis enzymes-Class 1 | - |
| GGPPS2 | LOC101490130 | Isoprenoid Biosynthesis enzymes-Class 1 | - |
| PSY1 | LOC101513117 | Isoprenoid Biosynthesis enzymes-Class 1 | - |
| PSY2 | LOC101501341 | Isoprenoid Biosynthesis enzymes-Class 1 | - |
| PSY3 | LOC101489077 | Isoprenoid Biosynthesis enzymes-Class 1 | - |
| PSY4 | LOC101509318 | Isoprenoid Biosynthesis enzymes-Class 1 | - |
| ZISO1 | LOC101501611 | NnrU | - |
| ZISO2 | LOC101502677 | NnrU | - |
| DXR1 | LOC101494630 | NADB_Rossmann | 1-deoxy-D-xylulose 5-phosphate reductoisomerase |
| DXR2 | LOC101490762 | NADB_Rossmann | 1-deoxy-D-xylulose 5-phosphate reductoisomerase |
| PDS | LOC101499699 | NADB_Rossmann | - |
| ZDS | LOC101493508 | NADB_Rossmann | - |
| CRTISO1 | LOC101500336 | NADB_Rossmann | - |
| CRTISO2 | LOC101494469 | NADB_Rossmann | - |
| LCYB | LOC101508316 | NADB_Rossmann | - |
| LEC | LOC101509804 | NADB_Rossmann | - |
| ZEP1 | LOC101503567 | NADB_Rossmann | Forkhead-associated |
| ZEP2 | LOC101492490 | NADB_Rossmann | Forkhead-associated |
| NSY | LOC101501945 | NADB_Rossmann | - |
| BCH1 | LOC101511972 | Fatty acid hydroxylase | - |
| BCH2 | LOC101509699 | Fatty acid hydroxylase | - |
| CYP97A | LOC101501150 | Cytochrome P450 | - |
| CYP97B | LOC101502085 | Cytochrome P450 | - |
| CYP97C | LOC101497194 | Cytochrome P450 | - |
| CCD1 | LOC101500703 | Retinal pigment epithelial membrane protein |  |
| VDE1 | LOC101494261 | Lipocalin / cytosolic fatty-acid binding protein | - |
| VDE2 | LOC101512965 | Lipocalin / cytosolic fatty-acid binding protein | - |

Table S3: Similarity matrices of DXS1-4 and PSY1-4 in chickpea based on the analysis using Bio Edit sequence alignment editor software (Hall, 1999).

| GENE ID | DXS1 | DXS2 | DXS3 | DXS4 |
| --- | --- | --- | --- | --- |
| DXS1 | 1 |  |  |  |
| DXS2 | 0.842 | 1 |  |  |
| DXS3 | 0.662 | 0.659 | 1 |  |
| DXS4 | 0.557 | 0.535 | 0.528 | 1 |
|  | | | | |
| GENE ID | PSY1 | PSY2 | PSY3 | PSY4 |
| PSY1 | 1 |  |  |  |
| PSY2 | 0.635 | 1 |  |  |
| PSY3 | 0.615 | 0.816 | 1 |  |
| PSY4 | 0.484 | 0.527 | 0.542 | 1 |

Table S4: The forward (F) and reverse (R) primer sequences of the 19 candidate genes including *Phytoene synthase (PSY1, PSY2, PSY3* and *PSY4), Phytoene desaturase synthase (PDS), 15-cis-zeta-carotene isomerase (ZISO1* and *ZISO2), ζ-carotene desaturase (ZDS), prolycopene isomerase (CRTISO1* and *CRTISO2), lycopene β-cyclase (LBC), lycopene ε-cyclase (LCYε), β-carotene hydroxylase (BCH1* and *BCH2), zeaxanthin epoxidase (ZEP1* and *ZEP2), violaxanthin de-epoxidase (VDE), crotenoid 9,10(9',10')-cleavage dioxygenase 1 (CCD1)* and *neoxanthin synthase (NSY)* involved in carotenoid biosynthesis and six housekeeping genes including *actin 1 (Act1), elongation factor 1-alpha (Ef1α), glyceraldehyde-3-phosphate dehydrogenase(GAPDH), initiation factor 4a (IF4a), heat shock protein 90 (HSP90)* and *18S ribosomal RNA (18SrRNA)* with their amplicon size.

| Gene name | Primer sequences | Amplicon length (bp) |
| --- | --- | --- |
| *PSY1* | F 5^´^-GGTGAGTGATTGTGCTCATTTG-3^´^  R 5^´^-CCAATCTCGATTCCCACCTATC-3^´^ | 112 |
| *PSY2* | F 5^´^-GCCCAAAGAGAATGCCAAATC-3^´^  R 5^´^-CCCACTTCCAAACTTTATCCTAGA-3^´^ | 97 |
| *PSY3* | F 5^´^-CACTTTGTGTATTGCAGTGTGG-3^´^  R 5^´^-TGGTGTGATGTGAGAAGCATTA-3^´^ | 76 |
| *PSY4* | F 5^´^-CCTTCCTTCACAAGGCTTATCT-3^´^  R 5^´^-GTCTGAACTGTACATGGCCTAA-3^´^ | 116 |
| *PDS* | F 5^´^-GCAGGACTGGCTGGTTTAT-3^´^  R 5^´^-ACCAGTCTCCATCTTCATCTTTC-3^´^ | 127 |
| *ZISO1* | F 5^´^-TGGCCGTCAAAGATTACCTAAA-3^´^  R 5^´^-CATAAGTGGGTGTGCAAAGTAAG-3^´^ | 106 |
| *ZISO2* | F 5^´^-ATTCCACTGGGTTCGGTAAG-3^´^  R 5^´^-ACTGTGGACACCAGCAAATA-3^´^ | 107 |
| *ZDS* | F 5^´^-GGGACCTGGTAAAGATCCATTT-3^´^  R 5^´^-CACCTTCCATGCTGTCTATGT-3^´^ | 107 |
| *CRTISO1* | F 5^´^-TGCCGTTGAAGAATGGAAGA-3^´^  R 5^´^-ACGCCTAAATCTCCACGAATAG-3^´^ | 99 |
| *RTISO2* | F 5^´^-TGTTATTGGGTCTGGGATTGG-3^´^  R 5^´^-CCAGAACTTCCACCAGGAATAA-3^´^ | 108 |
| *LCYB* | F 5^´^-GCTATTGACCCAAACCCTAGAT-3^´^  R 5^´^-CCAAGTTGTGTCAAGGCAATC-3^´^ | 102 |
| *LCYε* | F 5^´^-GTCGACTTACGGTAACGCTATT-3^´^  R 5^´^-CTCATCGGCGAAATCCTCTT-3^´^ | 100 |
| *crtRB1* | F 5^´^-GAACTCCGAGAGGTTCACTTAC-3^´^  R 5^´^-GAACTTCTCCACCCTCCATTT-3^´^ | 119 |
| *crtRB2* | F 5^´^-GTCCCATCATAGAGCAAGAGAAG-3^´^  R 5^´^-GATGAGTCCCTTGTGGAAGAAA-3^´^ | 115 |
| *ZEP1* | F 5^´^-GATGGTGCCTTCTTCGTAACT-3^´^  R 5^´^-GTGGACACGTGCAGGATAAT-3^´^ | 108 |
| *ZEP2* | F 5^´^-GATGATGCACTTGAGCGTACTA-3^´^  R 5^´^-GCCTCATCACGACTCAAACA-3^´^ | 101 |
| *VDE* | F 5^´^-GGAGAAGAAGGTAGAGGAAGGA-3^´^  R 5^´^-CCCTTCTGCCAACCTTTGTA-3^´^ | 118 |
| *CCD1* | F 5^´^-TGGCTCTGAGGCTGTTTATG-3^´^  R 5^´^-GCACGAATGATTTCCCGATATTC-3^´^ | 110 |
| *NSY* | F 5^´^-GCGGAACAAGTTTCCCTTTATG-3^´^  R 5^´^-TCATCCAACCACACACCATAG-3^´^ | 95 |
| *Act1* | F 5^´^-GCCTGATGGACAGGTGATCAC-3^´^  R 5^´^-GGAACAGGACCTCTGGACATCT-3^´^ | 62 |
| *Ef1α* | F 5^´^-TCCACCACTTGGTCGTTTTG-3^´^  R 5^´^-CTTAATGACACCGACAGCAACAG-3^´^ | 64 |
| *GAPDH* | F 5^´^-CCAAGGTCAAGATCGGAATCA-3^´^  R 5^´^-CAAAGCCACTCTAGCAACCAAA-3^´^ | 65 |
| *IF4a* | F 5^´^-TGGACCAGAACACTAGGGACATT-3^´^  R 5^´^-AAACACGGGAAGACCCAGAA-3^´^ | 60 |
| *HSP90* | F 5^´^-GCAGCATGGCTGGTTACATGT-3^´^  R 5^´^-TGATGGGATTCTCAGGGTTGA-3^´^ | 63 |
| *18SrRNA* | F 5^´^-ACGTCCCTGCCCTTTGTACAC-3´  R 5^´^-CACTTCACCGGACCATTCAAT-3^´^ | 61 |

Figure S1: The alignment of protein sequences of DXS1-4 (A) and PSY1-4 (B) in chickpea using Bio Edit sequence alignment editor software (Hall, 1999). Identical and similar amino acids are in black shaded and grey, respectively.

Figure S2: Expression pattern of carotenogenic genes including *Phytoene synthase 1 (*A), *Phytoene synthase 2* (B), *Phytoene synthase 3* (C), *Phytoene synthase 4* (D), *Phytoene desaturase synthase* (E), *15-cis-zeta-carotene isomerase 1* (F), *15-cis-zeta-carotene isomerase 1* (G), *ζ-carotene desaturase* (H*), carotene isomerase 1* (I), *carotene isomerase 2* (J), *lycopene β-cyclase* (*K*), *lycopene ε-cyclase* (L), *β-carotene hydroxylase 1* (M), *β-carotene hydroxylase 1* (N), *zeaxanthin epoxidase 1 (*O) *zeaxanthin epoxidase2* (P), *crotenoid 9,10(9',10')-cleavage dioxygenase 1* (Q) *violaxanthin de-epoxidase* (R), and *neoxanthin synthase* (S) in chickpea seeds at four developmental stages 8, 16, 24 and days post anthesis (DPA) in five cultivars CDC Frontier, CDC 441-34, CDC Verano, CDC Cory and CDC Jade
