# Supplementary figures and images for: Identification and Expression Analysis of Candidate Genes Involved in Carotenoid Biosynthesis in Chickpea Seeds
Source: Front Plant Sci. 2016 Dec 15;7:1867. doi: 10.3389/fpls.2016.01867 (PMC5157839; doi:10.3389/fpls.2016.01867)

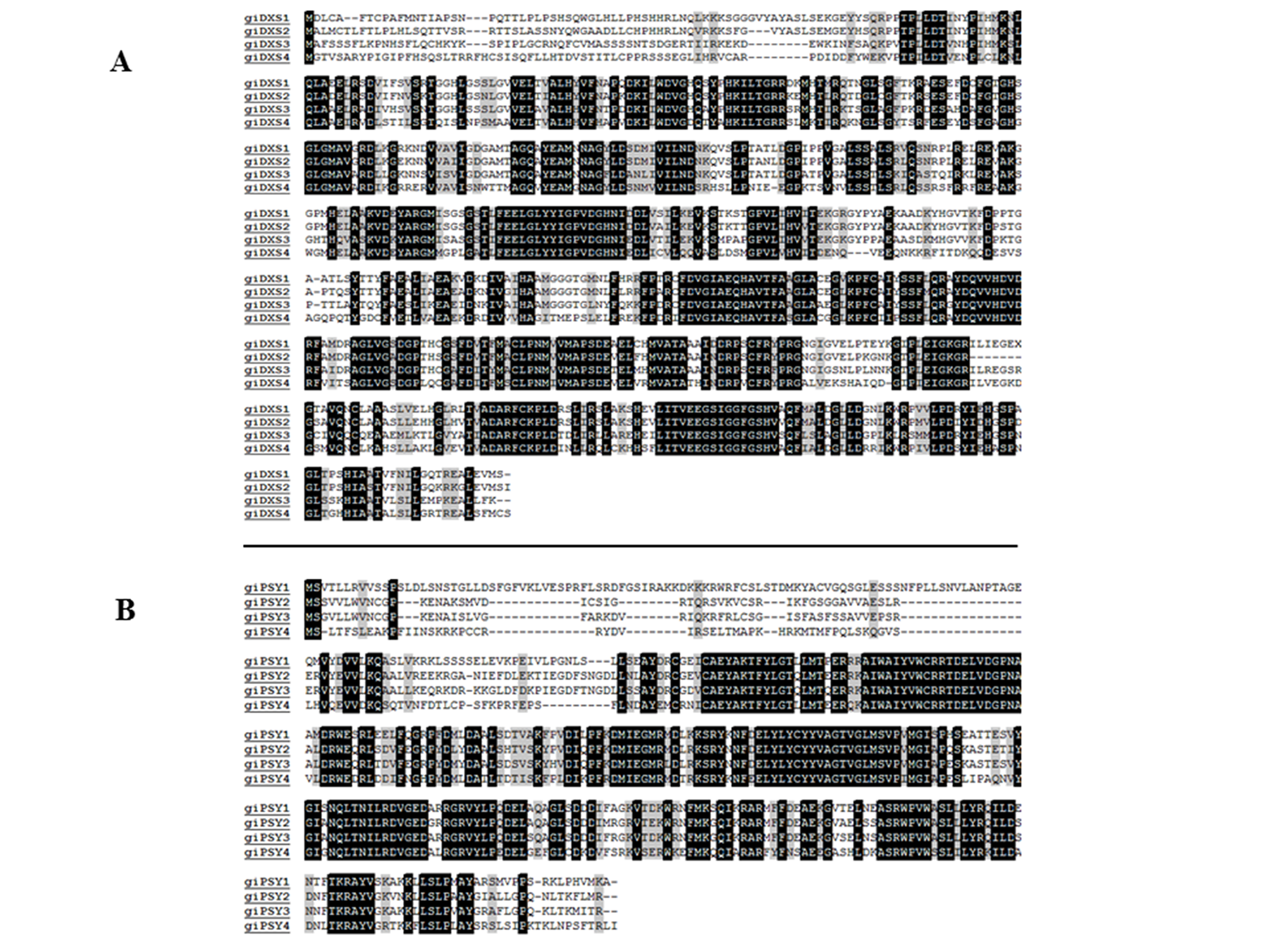

Supplement: Supplementary file 2 [file Image1.TIF]

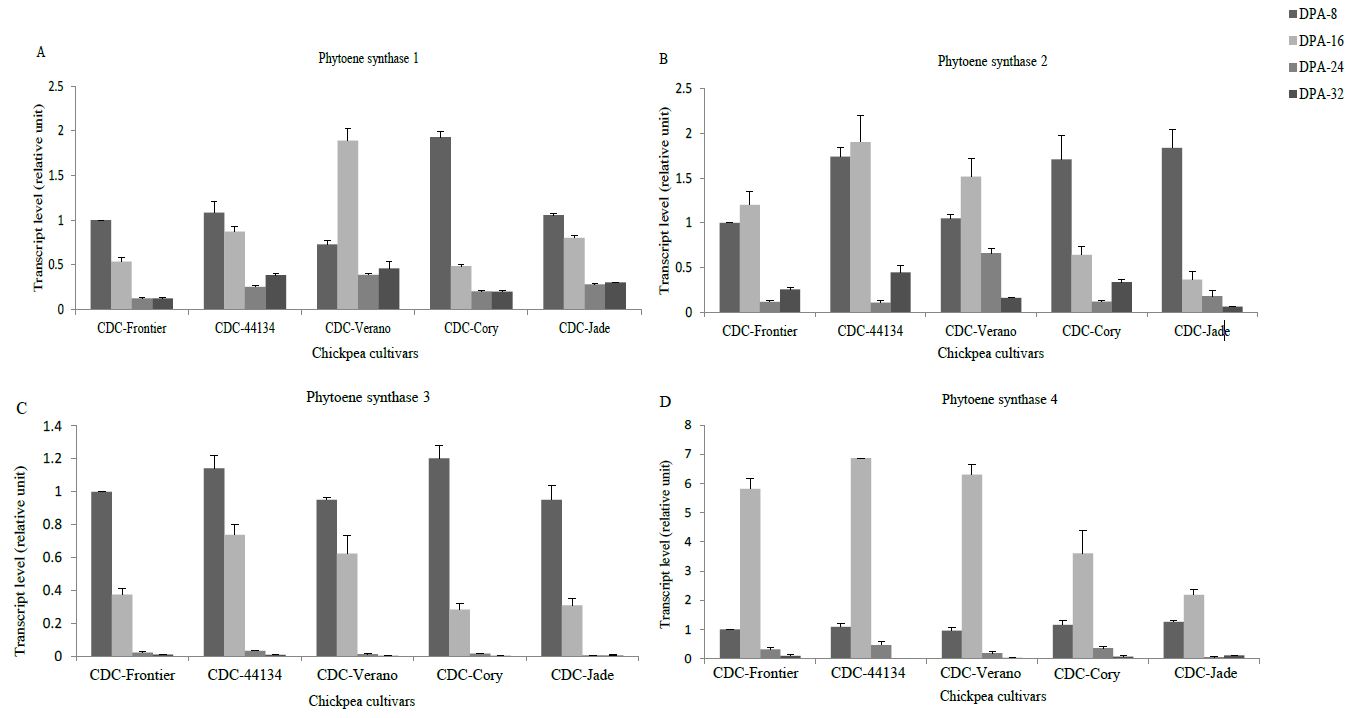

Supplement: Supplementary file 3 [file Image2.JPEG]

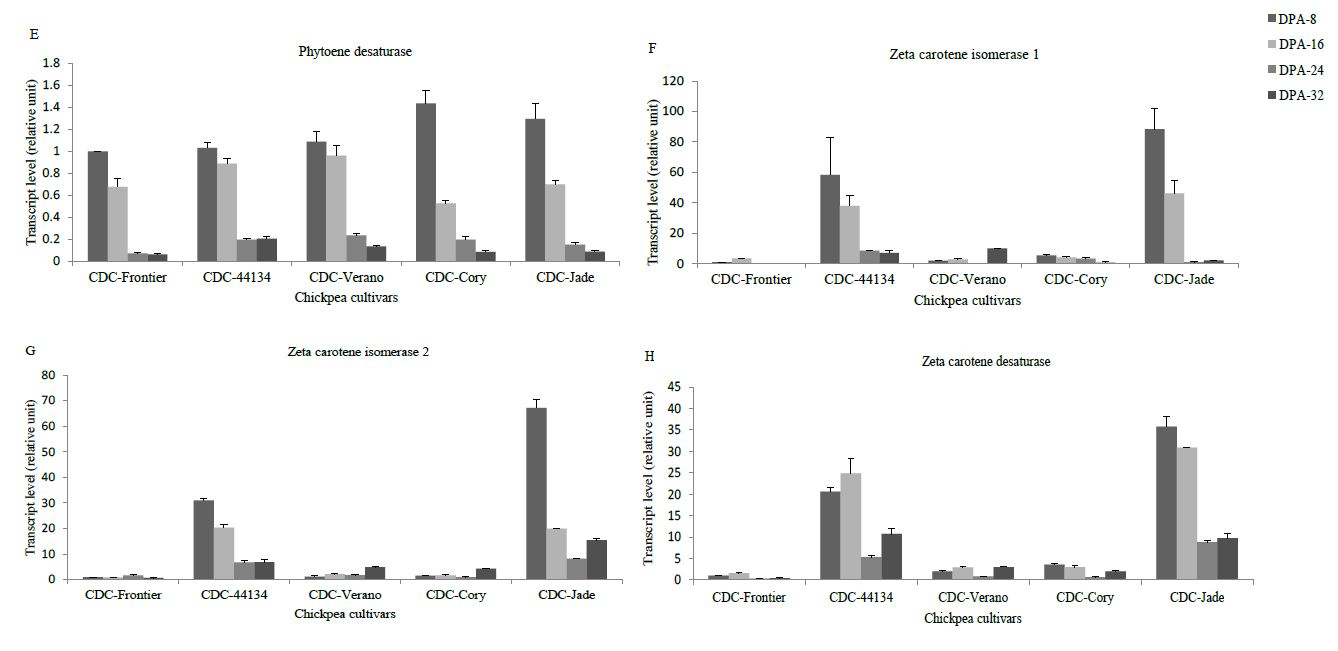

Supplement: Supplementary file 4 [file Image3.JPEG]

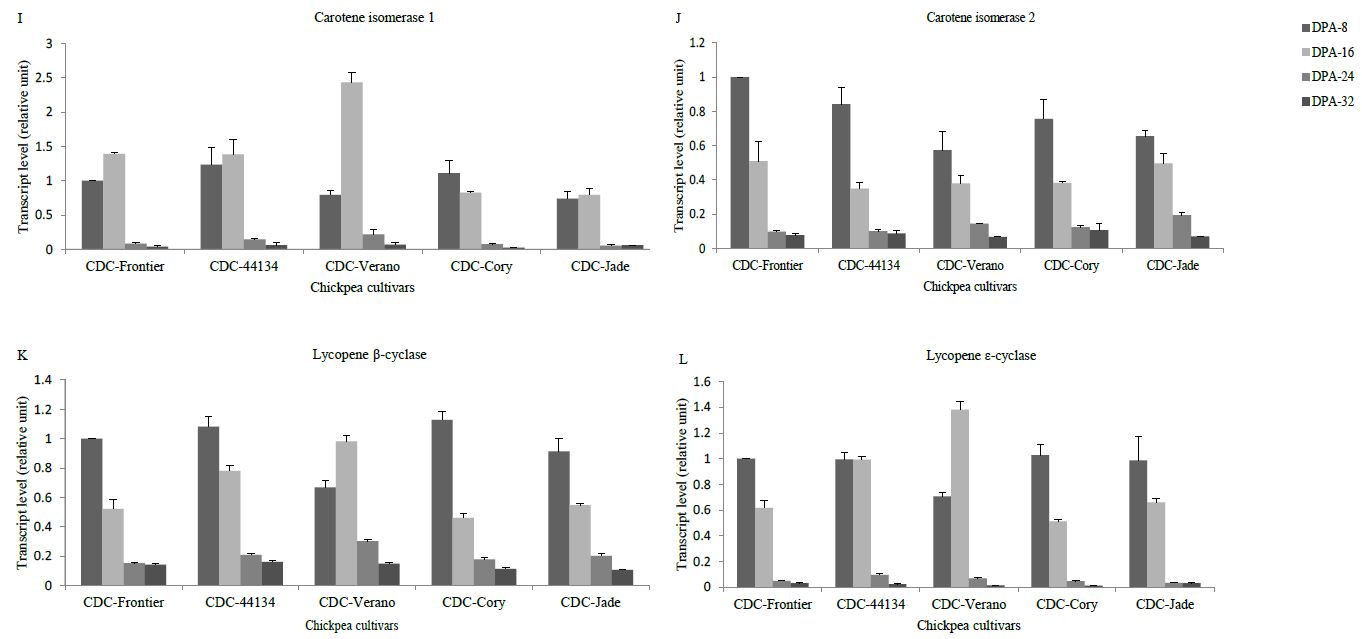

Supplement: Supplementary file 5 [file Image4.JPEG]

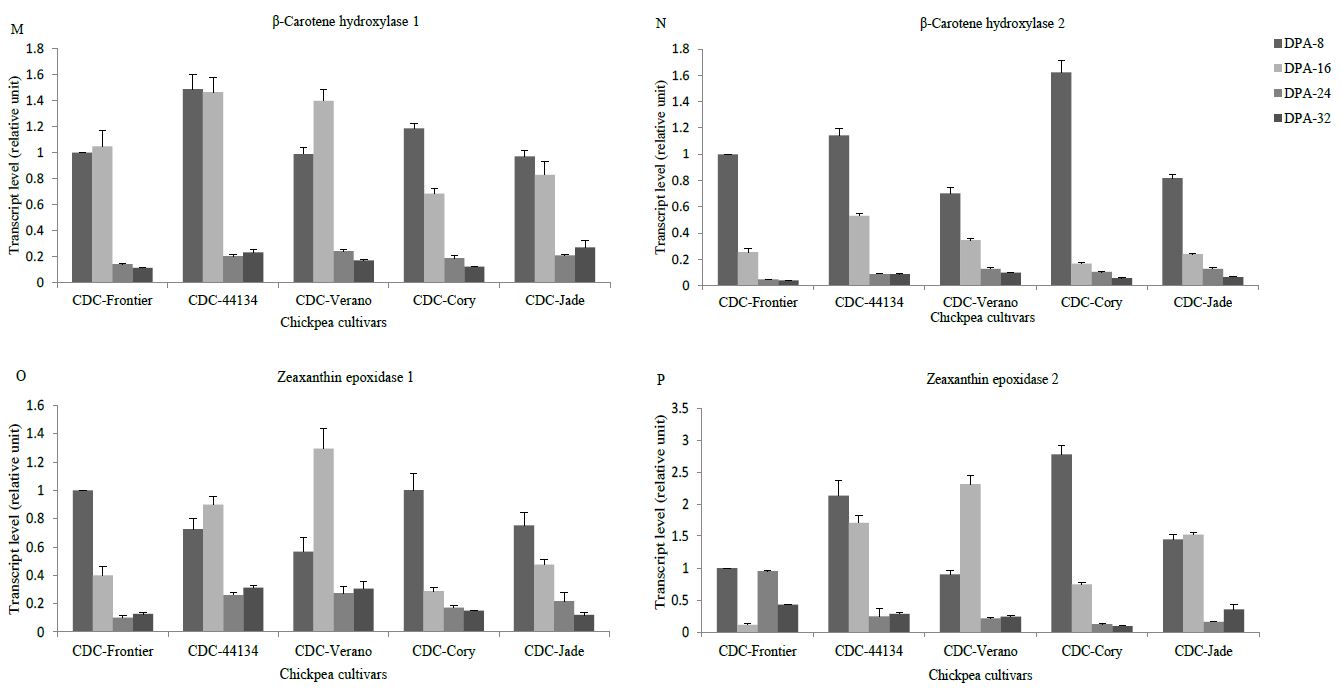

Supplement: Supplementary file 6 [file Image5.JPEG]

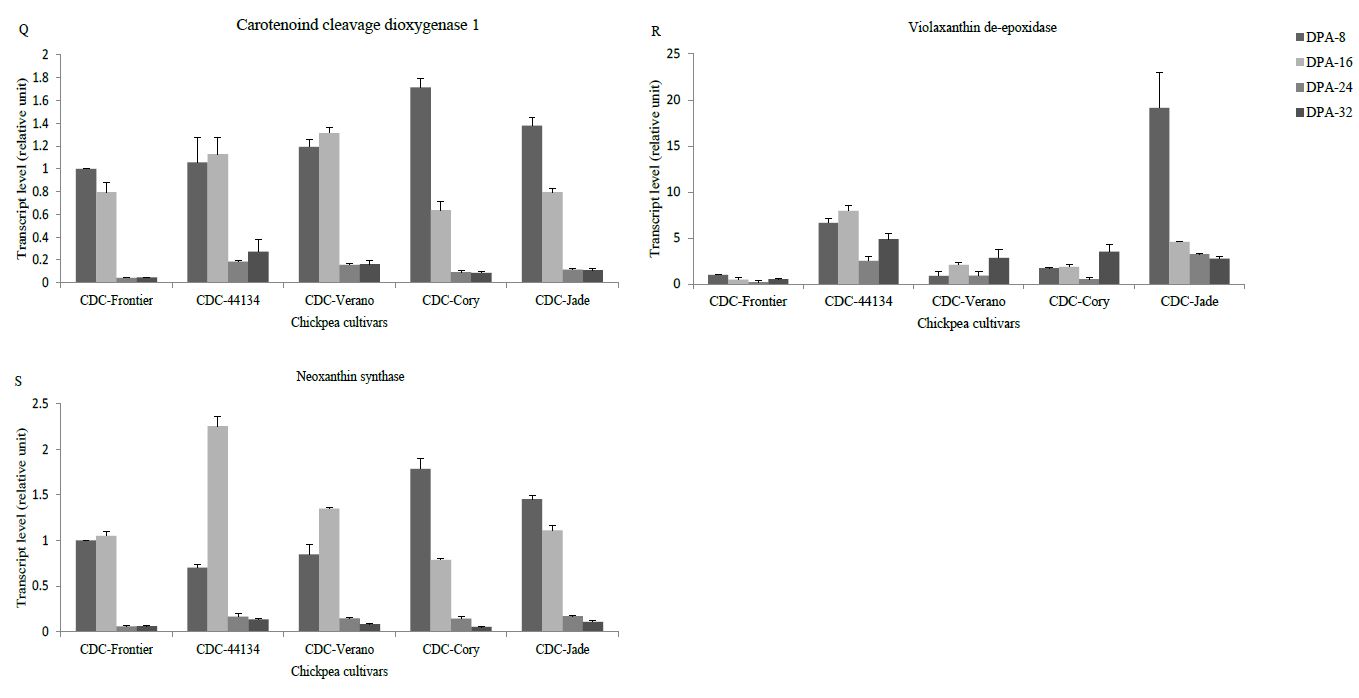

Supplement: Supplementary file 7 [file Image6.JPEG]
